# Supplementary material for: Nicotine Changes Airway Epithelial Phenotype and May Increase the SARS-COV-2 Infection Severity
Source: Molecules. 2020 Dec 28;26(1):101. doi: 10.3390/molecules26010101 (PMC7794754; doi:10.3390/molecules26010101)
Supplement: Supplementary file 1 [file molecules-26-00101-s001.pdf]

Supplementary Figures

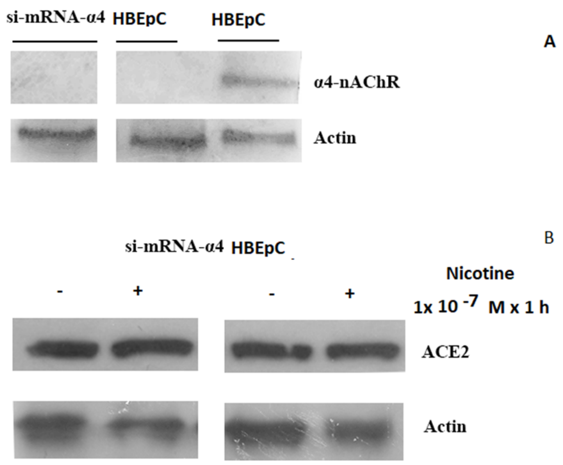

Figure S1. Expression of α4-nAChR and ACE2 in HBEpC in si-mRNA-α4-HBEpC after treatment for 1 h with Nicotine 1×10^{-7} M. **A:** Expression of α4-nAChR on wild type HBEpC and on si-mRNA-α4-HBEpC. **B** expression of ACE2.

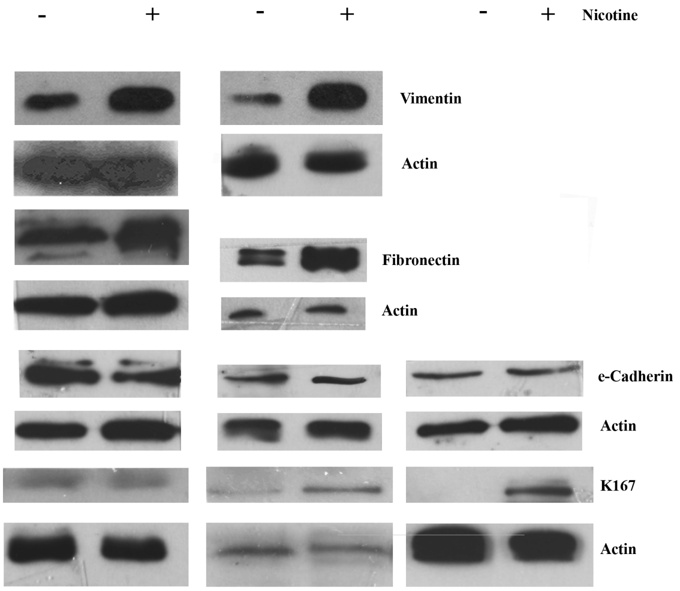

Figure S2. Western blot raw data.

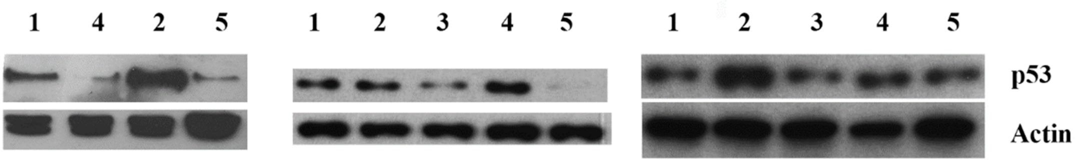

1 CTRL

2 positive control H_2O_2 400 μM for 1 h

3. Nicotine 1 x 10^{-7} M for 1 h

4 Nicotine 1 x 10^{-7} M for 24 h

5 Nicotine 1 x 10^{-7} M for 48 h
